# Particular *Candida albicans* Strains in the Digestive Tract of Dyspeptic Patients, Identified by Multilocus Sequence Typing

**DOI:** 10.1371/journal.pone.0035311

**Published:** 2012-04-20

**Authors:** Yan-Bing Gong, Jian-Ling Zheng, Bo Jin, De-Xiang Zhuo, Zhu-Qing Huang, He Qi, Wei Zhang, Wei Duan, Ji-Ting Fu, Chui-Jie Wang, Ze-Bin Mao

**Affiliations:** 1 Laboratory of Ze-Bin Mao, Department of Biochemistry and Molecular Biology, School of Basic Medical Sciences, Peking University Health Science Center, Beijing, China; 2 Department of Microbiology, Medical Sciences Institute of Liaoning, Shenyang, China; 3 Division of Gastroenterology, The First Hospital of Liaoning University of Traditional Chinese Medicine, Shenyang, China; University of Aberdeen, United Kingdom

## Abstract

**Background:**

*Candida albicans* is a human commensal that is also responsible for chronic gastritis and peptic ulcerous disease. Little is known about the genetic profiles of the *C. albicans* strains in the digestive tract of dyspeptic patients. The aim of this study was to evaluate the prevalence, diversity, and genetic profiles among *C. albicans* isolates recovered from natural colonization of the digestive tract in the dyspeptic patients.

**Methods and Findings:**

Oral swab samples (*n* = 111) and gastric mucosa samples (*n* = 102) were obtained from a group of patients who presented dyspeptic symptoms or ulcer complaints. Oral swab samples (*n* = 162) were also obtained from healthy volunteers. *C. albicans* isolates were characterized and analyzed by multilocus sequence typing. The prevalence of *Candida* spp. in the oral samples was not significantly different between the dyspeptic group and the healthy group (36.0%, 40/111 *vs.* 29.6%, 48/162; *P* > 0.05). However, there were significant differences between the groups in the distribution of species isolated and the genotypes of the *C. albicans* isolates. *C. albicans* was isolated from 97.8% of the *Candida*-positive subjects in the dyspeptic group, but from only 56.3% in the healthy group (*P* < 0.001). DST1593 was the dominant *C. albicans* genotype from the digestive tract of the dyspeptic group (60%, 27/45), but not the healthy group (14.8%, 4/27) (*P* < 0.001).

**Conclusions:**

Our data suggest a possible link between particular *C. albicans* strain genotypes and the host microenvironment. Positivity for particular *C. albicans* genotypes could signify susceptibility to dyspepsia.

## Introduction

*Candida* is considered part of the normal human flora, but recently, much attention has been given to the role of *Candida* in chronic gastritis and ulcerous disease. *Candida albicans* (*C. albicans*) colonization of the gastric mucosa was shown to be present in 30–50% of patients with active ulcerous disease [1]–[5]. The presence of *C. albicans* prolongs the persistency of clinical symptoms and affects the processes of ulcer healing [6]–[8].

*C. albicans* can invade epithelial cells by induced endocytosis and/or active penetration. The routes of adhesion, invasion, and damage by *C. albicans* depend not only on fungal morphology and activity, but also on the epithelial cell type and the differentiation stage of the epithelial cells, indicating that epithelial cells differ in their susceptibility to the fungus. The ability of *Candida* to invade different environments in the host organism is a result of the great flexibility and adaptability of fungi [9]–[15]. The isolation and identification of *C. albicans* strains involved in lesions of the gastric mucosa would contribute to an understanding of the relationship between *C. albicans* and inflammatory gastrointestinal barrier disorders.

Molecular epidemiology studies have shown that *C. albicans* isolates exhibit a high level of genetic diversity. Multilocus sequence typing (MLST) has been used to discriminate *C. albicans* strains and to detect small genetic changes or microvariations that may be indicative of adaptability processes [16]–[41]. MLST of *C. albicans* is a highly discriminatory method based on the analysis of nucleotide polymorphisms within the sequences of seven PCR-generated 400- to 500-bp internal fragments of housekeeping genes (the loci). For each locus, the different sequences are assigned to distinct genotypes (represented by integers), and for each isolate, the combination of the genotypes at each of the sequenced loci defines a profile referred to as the diploid sequence type (DST). Because MLST relies only on nucleotide sequencing, it generates highly standardized data that can be exchanged through a web-based database (http://calbicans.mlst.net).

Genetic profiles of the *C. albicans* strains in the digestive tract of dyspeptic patients have not been investigated before. In the present work, we assessed isolates of *C. albicans* from the gastric and oral mucosa by MLST, to ascertain whether particular strains are related to pathological lesions of the human gastric mucosa. Our results showed that the DST1593 was the dominant genotype in the digestive tract of dyspeptic patients; this could be used as a sign of susceptibility to dyspepsia.

## Results

### Prevalence of *Candida* in Oral Swab Samples from Healthy People

The healthy group comprised 162 subjects from 32 families from Shenyang, China. Forty-eight subjects were colonized by *Candida* species. The majority of people (95.8%) were colonized by only one species. *C. albicans* was identified in 56.3% of the carriers, *C. parapsilosis* was identified in 20.8% of the carriers, *C. tropicalis* in 10.4%, *Meyerozyma guilliermondii* in 6.3%, *Lodderomyces elongisporus* in 4.2%, and other *Candida* species (including *C. metapsilosis*, *Issatchenkia orientalis*, and *C. ethanolica*) were identified in 6.3% of the carriers. *C. albicans* carriers were identified in 18 families. Overall, the rate of oral carriage for *C. albicans* was 16.7%.

### Prevalence of *Candida* in Oral Swabs and Gastric Mucosa Samples from People with Dyspepsia

The dyspeptic group comprised 111 dyspeptic patients, and the family control group consisted of one or two family members for each of 18 patients. Forty-six dyspeptic patients were colonized by *Candida* species. The majority of the dyspeptic patients (95.7%) were colonized by only one species. Twenty-four patients were colonized at only one of the two sites studied (oral [18 patients] and gastric mucosa [six patients]), whereas 22 patients were colonized at both sites. *C. albicans* was identified in all but one of the 46 *Candida* carriers; the other was positive for *C. glabrata*. *C. parapsilosis* was identified in 4.3% of the *Candida* carriers and *C. glabrata* was identified in 2.2% of the *Candida* carriers. Overall, the rate of oral and/or gastric carriage for *C. albicans* was 40.5% in the dyspeptic group. *C. albicans*, and no other species, was present in oral swabs from four of the family control group: three were patients’ wives and one was a patient’s daughter.

There was a statistically significantly increased prevalence of *C. albicans* in oral swabs from the dyspeptic group *versus* the healthy group (34.2% *vs.* 16.7%, respectively; *P*  =  0.001). The prevalence of *Candida* spp. in oral swabs was not significantly different between the two groups (36.0% *vs.* 29.6%; *P* > 0.05) (Table 1).

**Table 1:** The prevalence of *Candida* spp. and *C. albicans* in oral swab samples from the dyspeptic group and the healthy group.

Type of subject	No. of subjects	Oral swab positivity, *n* (%)			
		*Candida* spp.		*C. albicans*	
Dyspeptic patients	111	40	(36.0)	38	(34.2)
Healthy subjects	162	48	(29.6)	27	(16.7)
Total	273	88	(32.2)	65	(23.8)

### MLST Analysis of the *C. albicans* Isolates

Taken together, 96 *C. albicans* samples were isolated: 27 from gastric mucosa samples of the dyspeptic patients and 69 from oral swab samples (from 38 patients, 4 patient family members, and 27 healthy volunteers). The samples were grown in dishes and two clones from each dish were chosen for MLST analysis. All but 11 of the replicates shared the same DST; those that were different were from two gastric mucosa samples (nos. 8027 and 8066), four oral swabs from the dyspeptic patients (nos. 8027, 8113, 8203, and 8221), and five oral swabs from the healthy volunteers (nos. 2n, 42a, 157c, 287, and 287g). Details of the *Candida* prevalence in the healthy volunteers and the dyspeptic group are presented in Tables S1 and S2, respectively. As shown in Tables S1 and S2, 46 different DSTs were identified: 37 were identified in a single sample and nine were shared by multiple samples (DST142, DST367, DST601, DST656, DST1593, DST1594, DST1779, DST1957, and DST1971). Twenty-nine novel DSTs (DST1593, DST1594, and DST1956–1978) were submitted to the MLST database (http://calbicans.mlst.net). Their details are presented in Table S3.

**Figure 1:**
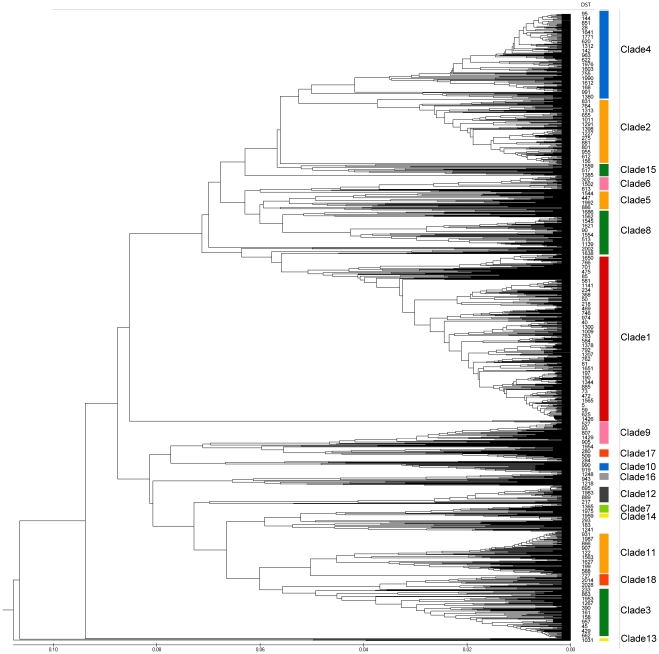
UPGMA dendrogram for *C. albicans* DSTs in the web-based database. The 18 clades and some DSTs are indicated.

To investigate the evolutionary relationships between the DSTs isolated in our study, we performed an eBURST analysis and constructed a dendrogram based on the unweighted-pair group method using average linkages (UPGMA) on all the *C. albicans* DST data in the database (Table S4 and Figure 1). The DSTs were divided into 18 clades in the UPGMA dendrogram [30], or 103 groups in eBURST analysis, plus singletons. Two DSTs (DST1957 and DST1978) isolated in our study were singletons, and the others were distributed among 11 clades.

Overall, DST1593 (in clade 18) was the most common genotype: it was shared by 32 of the 76 *C. albicans* carriers (27 dyspeptic patients, one wife of a dyspeptic patient, and four healthy subjects). However, within the groups, DST1593 was most common only in the dyspeptic group, in which it comprised 60% (27/45) of *C. albicans* carriers (in oral swabs from six subjects, gastric mucosa from nine subjects, and both sites from 12 subjects) compared with only 14.8% (4/27) of *C. albicans* carriers in the healthy group (*P* < 0.001). The other DSTs in clade 18 (DST1594, DST1958, DST1970, and DST1971) were all isolated from the dyspeptic group.

We next compared the DSTs between the three dyspeptic patients whose wives were also positive for *C. albicans*. In only one family, no. 8063, the wife shared the DST1593 *C. albicans* genotype with her husband who suffered from superficial gastritis; their daughter was negative for *Candida*. Meanwhile, in the other two families, different DSTs in different clades were isolated between the dyspeptic patients and their wives (DST1593 *versus* DST1957 in family no. 703 and DST1779 *versus* DST1969 in family no. 709). The rate of intrafamilial transmission of the same DST was only 5.6% (1/18).

**Figure 2:**
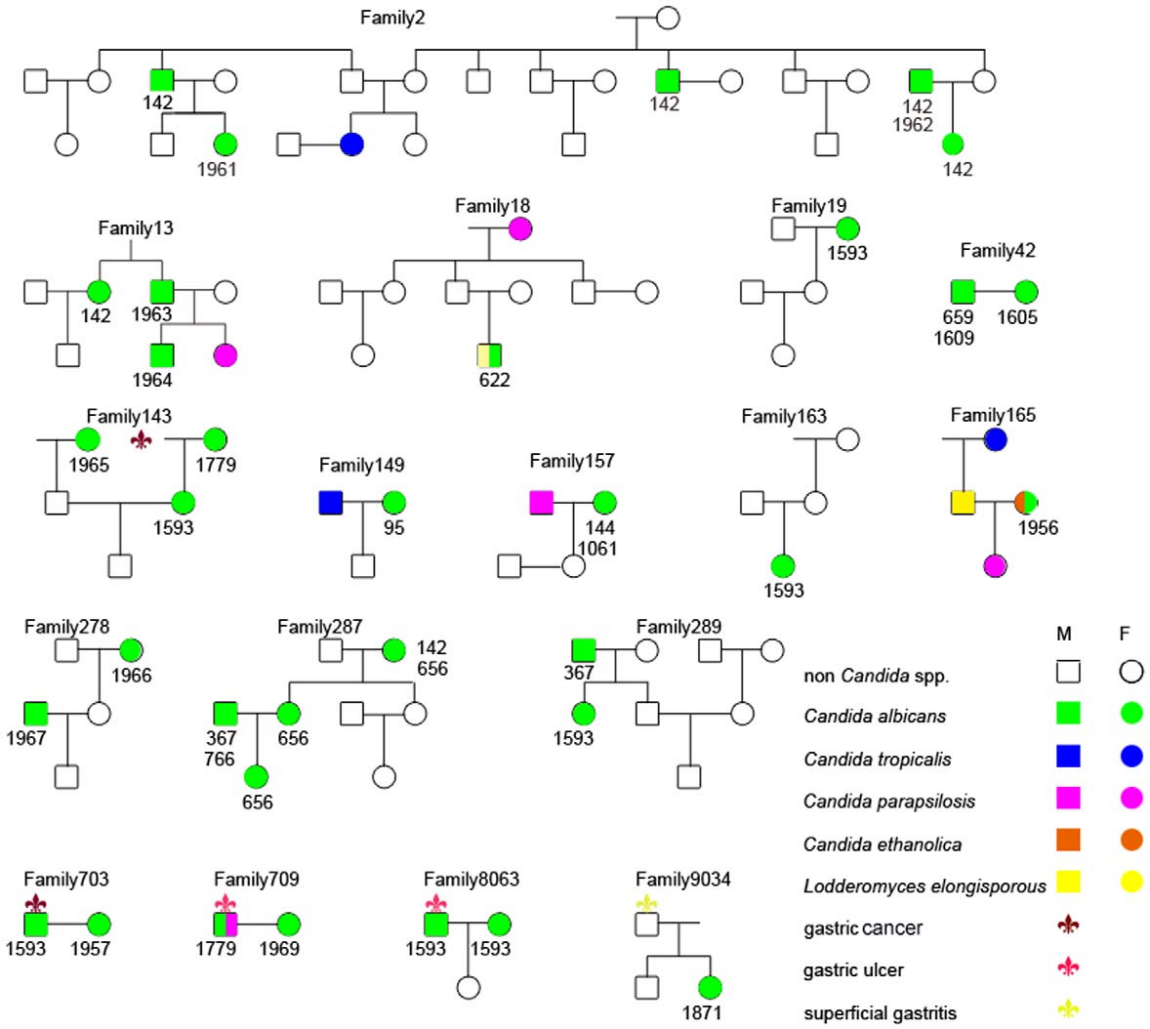
Distribution of DSTs among the families positive for *C. albicans*, including those containing dyspeptic patients. The number under the green square or circle is the DST no.

DST142 (in clade 4) was the second most common genotype among all the *C. albicans* isolates, and the most common genotype in the healthy group. It was isolated from seven subjects (six from oral swabs from the healthy group and one from an oral swab from the dyspeptic group). Four of the seven DST142 carriers were cousins in family no. 2. In another family positive for DST142, no. 287, the dominant genotype was DST656 (in clade 4), which was identified in three bloodline relatives (the grandmother [positive for DST142 and DST656], the mother, and a female baby), whereas the father was positive for DST367 and DST766 (in clade 1). The other DST142-carriers were from family no. 13 and patient no. 516. Although they lived in the same province, albeit a different region within it, these four DST142-positive families would have little chance of encountering each other. DST1605 and DST1609 (in clade 4) were isolated from family no. 42; DST1966 and DST1967 (in clade 5) were isolated from family no. 278. Both sets of genotypes have a very close evolutionary relationship, suggesting that intrafamilial transmission might have occurred. Therefore, intrafamilial transmission may have occurred in four of the eighteen families (nos. 2, 42, 278, and 287; a rate of 22.2%). The distribution of DSTs is presented in Figure 2.

DST1779 (in clade 15) was isolated from two patient gastric mucosa samples and one healthy control oral sample. Similar to DST1593, DST1779 has an intimate link to the dyspeptic group. In family no. 143, the wife carried DST1593, the wife’s mother, who suffered from hyperthyreosis and dry mouth, was positive for DST1779, the wife’s late father died ten years ago from gastric cancer, and the husband’s mother carried DST1965 (in clade 3). The husband and the son were not positive for any *Candida* spp. In contrast to families nos. 2 and 287, different DSTs in different clades were isolated from family no. 143.

Both carriers of DST367 (in clade 1) (nos. 287 and 289g) were from the healthy group, and, being from different families and different regions, had little chance of encountering each other. DST1957 (a singleton) was isolated from an oral sample from a dyspeptic patient (no. 422a) and the wife (no. 703b) of another patient. Both DST1957 carriers attended the clinic on different dates, and were from different families and different regions. Thus, it is unlikely that this DST became shared by different subjects by nosocomial infection.

In this study, the most common strain group was clade 18, which was shared by 35 subjects among all the 76 *C. albicans* carriers (46.1%), followed by clade 4 (25%), clade 14 (6.6%), clade 15 (6.6%), clade 5 (5.3%), clade 1 (3.9%), singletons (3.9%), clade 3 (2.6%), clade 7 (2.6%), clade 8 (2.6%), clade 9 (1.3%), and clade 12 (1.3%). Most *C. albicans* isolates in the oral and gastric mucosa samples from a single subject were in the same clade, except the fourteen isolates from six patients that were in different clades: no. 8066 (clades 4 and 18), no. 8113 (clades 7 and 18), no. 8115 (clades 4 and 18), no. 8151 (clades 15 and 18), no. 8184 (clades 8 and 18), and no. 8203 (clades 14 and 18). Most strains in the healthy group were in clade 4 (48.1%); most strains in the dyspeptic group were in clade 18 (66.7%). The strains in clades 1 and 3 were not isolated from the dyspeptic group; the strains in clades 7, 8, 9, 12, and 14 were not isolated from the healthy group.

## Discussion

The aim of this study was to evaluate the prevalence, diversity, and genetic profiles among *C. albicans* isolates recovered from natural colonization of the digestive tract in dyspeptic patients. We screened oral and/or gastric mucosa samples from 111 subjects with dyspeptic symptoms, and oral samples from 162 healthy volunteers for the presence of *Candida* spp. Few *Candida* species were identified in the dyspeptic group, among which *C. albicans* was present in the overwhelming majority of positive cases in the dyspeptic group (97.8%), but only just over half of positive cases in the healthy group (56.3%). Furthermore, there was a significant difference in the species distribution and the *C. albicans* genotypes between the dyspeptic patients and the healthy volunteers. The low pH in the stomach may be the primary factor for *C. albicans* being the dominant *Candida* species in the gastric mucosa. *C. albicans* can grow well in an environment of pH  =  2, while most other *Candida* species cannot [3], [42], [43]. It puzzled us why *C. albicans* was also the dominant *Candida* species in the oral environment of the dyspeptic patients, where the pH is neutral. This needs further study.

We used MLST to evaluate the diversity and genetic relationships among the 192 *C. albicans* isolates recovered from 96 samples. A significant number of isolates with identical DSTs were observed in our study: for instance, DST1593 was found in 60% of *C. albicans*-positive dyspeptic patients but in only 14.8% of healthy *C. albicans* carriers. DST1593 was not isolated from any of the operators of this study; therefore, nosocomial infection cannot explain the higher frequency of DST1593 among the dyspeptic patients.

Strains in clade 18 were isolated from 66.7% of *C. albicans*-carriers in the dyspeptic group. Meanwhile, the strains in clade 4, the second most common clade worldwide, were found mainly in the healthy group (48.1%). This observation is consistent with differences in the host microenvironment being a primary factor in determining the different clade strain distributions between healthy individuals and dyspeptic patients.

According to Sampaio *et al.* [17], *C. albicans* genetic variants of the same strain undergo adaptations to environmental changes in the host. Adherence to the adhesion molecules of the epithelial cells by the *C. albicans* adhesins, and adaptation to the microenvironment of the host, were shown to be the most important determinants of whether the *C. albicans* strains were able to colonize the host. Thus, the DST1593 *C. albicans* strains might be the best suited to the mucosa of the dyspeptic patients, and the presence of DST1593 could be a sign of susceptibility to dyspepsia.

Another important factor in colonization of the host is whether the *C. albicans* strains are tolerated by the host’s immune system. In this study, DST1779 was likely to be a sign of a complicated mix of both tolerance and recognition/attack by the immune system. DST1779 was firstly reported from a patient with APECED (autoimmune polyendocrinopathy-candidiasis-ectodermal dystrophy) in Ireland by McManus *et al.* [44]. In this study, one of the three DST1779-carriers suffered from hyperthyreosis and dry mouth (an autoimmune disease), while the other two carriers had atrophic gastritis and a gastric ulcer, respectively. Peptic ulcers have often been reported to have an intricate relationship with autoimmune disease [45]–[47]. DST1779 is an uncommon *C. albicans* genotype worldwide, and there is little chance of transmission between the carriers in Shenyang and Ireland. This suggests, therefore, that DST1779 might be a sign of autoimmune disease in hosts sharing a similar microenvironment. This is worthy of further study.

Wrobel *et al.* demonstrated that *C. albicans* is associated mainly with humans, and environmental *C. albicans* isolates are rare [24]. Their data suggest a great likelihood of *C. albicans* person-to-person transmission, and the effect of global human travel is likely to reduce geographically separated evolutionary trends [23]. According to the literature, the geographic specificity that has been proposed for the different clades should be reconsidered [20], [23], [30], [32], [35], [48]–[51]. In the web-based database, the strains in clades 2, 6, and 10 have not been reported in the Greater China area. In this study, 77.8% of strains from the healthy group were in clades 1, 3, 4, or 5, and the strains in these four clades lack geographic or ethnic differences. Meanwhile, 71.1% of strains from the dyspeptic group were in clades 14 or 18; all strains in these two clades have been previously reported from the west coast of the Pacific Ocean (China, Japan, Korea, and Australia), except for one case in the UK. It seems that geographic or ethnic differences might play a minor role for the strains in the healthy group, but a major role for the strains in the dyspeptic group. It is unknown whether DST1593 will be isolated from dyspeptic patients of all areas and races, or only from Asians. This should be tested in different populations.

In summary, the data presented in this study indicate that the prevalence of commensal *Candida* and the distribution of the *Candida* species isolated are different between dyspeptic and healthy individuals. We indicated a link between DST1593 and dyspepsia; this genotype could be a sign of susceptibility to dyspepsia.

Future studies should aim to reveal the nature and molecular basis of these microevolutions in strain, and to evaluate their impact on the fitness of *C. albicans* isolates in the context of the host mucosa. It would also be interesting to investigate to what degree the DST1593 *C. albicans* genotype influences susceptibility to dyspepsia among different ethnic or geographical populations.

## Materials and Methods

### Ethics Statement

All samples were taken with written informed consent from all study participants or the parents of the minors. This study was approved by the Peking University Institutional Review Board.

### Specimen Collection

Oral swab samples (*n*  =  111) and human gastric mucosa lesion samples (*n*  =  102) were obtained from a group of 111 dyspeptic patients, comprising 39 females and 72 males with a mean age of 47.6 years (median: 46.0 years; range: 18–80). They presented dyspeptic symptoms or ulcer complaints, involving 77 with chronic gastritis, 29 with a peptic ulcer, and five with gastric cancer. The gastric mucosa samples were collected by gastroscopy with a biopsy for mycological, *Helicobacter pylori* (*H. pylori*), and histological evaluation. Patients receiving chemotherapy, and also those who had been treated with steroids or antibiotics before the study, were excluded. Clinical examination included taking a medical history and gastroscopy with biopsy.

Oral swab samples (*n*  =  21) were obtained from volunteers from 18 of the patients’ family members as a family control group. The samples were provided anonymously, and comprised thirteen samples from the spouses of thirteen patients, six from the children of six patients, one from the grandson of one patient, and one from the mother of one patient. All cases were ascertained in the Division of Gastroenterology at the First Hospital of Liaoning University of Traditional Chinese Medicine in Shenyang City, China, between July 10^th^ and September 12^th^, 2008.

Oral swab samples (*n*  =  162) were also obtained from healthy volunteers who had no dyspeptic complaint, comprising 92 females and 70 males with a mean age of 39.47 years (median: 39.50 years; range: 4–81). The volunteers were from 32 families (*i.e.*, the parents and the first and second generations of their children) of the students or employees of Liaoning University of Traditional Chinese Medicine in Shenyang City, China. The samples were taken between May 1^st^ and October 29^th^, 2008.

### Culture, Identification, MLST, and Analysis of *Candida*

The oral swab or gastric mucosa samples from each subject were plated on Sabouraud agar (Tianhe Microorganism Reagent Co., Ltd., Hangzhou, China) and incubated at 37°C for 48 h. Yeast colonies were streaked onto CHROM agar *Candida* medium (CHROM agar Microbiology, Paris, France) and incubated at 37°C for 48 h. Green colonies were presumed to be *C. albicans* and were re-streaked for purification. Two clones from each dish were picked for further analysis and storage. Verification that the isolate was *C. albicans* was accomplished using PCR with primers that amplified the ITS1–5.8S–ITS2 region of the ribosome-encoding DNA [52]–[54]. All *C. albicans* colonies were further typed by MLST as described previously [40], [41]. The MLST data were assigned to genotypes for the seven loci sequenced and to DSTs by reference to the *C. albicans* MLST database (http://calbicans.mlst.net). The genetic relatedness between the investigated strains was evaluated by constructing a UPGMA with arithmetic averages dendrogram using MEGA software (http://www.megasoftware.net/), which compares genotypic profiles [30]. The DST data were also analyzed by eBURST (http://calbicans.mlst.net/eburst/) to investigate the evolutionary relationships among all the *C. albicans* strains in the database.

### Statistical Analysis

The statistical significance of the results was determined by the Pearson chi-squared test, using SPSS version 11.5 (SPSS, Inc., Chicago, IL). The results were considered statistically significant with *P* values of less than 0.05.

## Supporting Information

Table S1 **Details of the** ***Candida*** **positivity in the healthy group.**
(DOC)

Table S2 **Details of the** ***Candida*** **positivity in the dyspeptic group and their family members.**
(DOC)

Table S3 **Details of the seven** ***C. albicans*** **gene loci for DST1593–1978.**
(DOC)

Table S4 **The UPGMA dendrogram for all the** ***C. albicans*** **DSTs in the web-based database.**
(XLS)
