# Supplementary material for: Particular Candida albicans Strains in the Digestive Tract of Dyspeptic Patients, Identified by Multilocus Sequence Typing
Source: PLoS One. 2012 Apr 20;7(4):e35311. doi: 10.1371/journal.pone.0035311 (PMC3335024; doi:10.1371/journal.pone.0035311)
Supplement: Table S1 — Details of the Candida positivity in the healthy group. (DOC) [file pone.0035311.s001.doc]

**Table S1.** Details of the *Candida* positivity in the healthy group

| Family  no. | subject | isolate | age | sex ^a^ | species | ITS ^b^ | DST ^c^ | CLADE | eBURST  group |
| --- | --- | --- | --- | --- | --- | --- | --- | --- | --- |
| 2 | 2c | ZA001 | 28 | F | *C. tropicalis* | FJ697166 | - | - | - |
|  | 2f | ZA002 | 65 | M | *C. albicans* | FJ697167 | 142 | 4 | 2 |
|  | 2h | ZA003 | 36 | F | *C. albicans* | FJ619278 | 1961 | 3 | 4 |
|  | 2n | ZA004 | 41 | M | *C. albicans* | FJ662393 | 1962 | 4 | 2 |
|  |  | ZA004a |  |  | *C. albicans* | JN606247 | 142 | 4 | 2 |
|  | 2o | ZA005 | 18 | F | *C. albicans* | FJ662391 | 142 | 4 | 2 |
|  | 2t | ZA006 | 43 | M | *C. albicans* | FJ662398 | 142 | 4 | 2 |
| 13 | 13 | ZA007 | 22 | F | *C. parapsilosis* | FJ662411 | - | - | - |
|  | 13a | ZA008 | 45 | M | *C. albicans* | FJ662407 | 1963 | 1 | 9 |
|  | 13c | ZA010 | 10 | M | *C. albicans* | FJ662405 | 1964 | 5 | 76 |
|  | 13f | ZA011 | 48 | F | *C. albicans* | FJ662404 | 142 | 4 | 2 |
| 18 | 18b | ZA012 | 70 | F | *C. parapsilosis* | FJ662412 | - | - | - |
|  | 18g | ZA013 | 5 | M | *C. albicans* | FJ662397 | 622 | 4 | 2 |
|  |  | ZA013a |  |  | *Lodderomyces elongisporus* | JN606251 | - | - | - |
| 19 | 19c | ZA014 | 56 | F | *C. albicans* | JN606252 | 1593 | 18 | 10 |
| 21 | 21 | ZA015 | 38 | F | *C. parapsilosis* | FJ697170 | - | - | - |
|  | 21a | ZA016 | 39 | M | *Meyerozyma guilliermondii* | GQ280287 | - | - | - |
|  | 21b | ZA017 | 8 | M | *C. parapsilosis* | GQ280288 | - | - | - |
|  | 21g | ZA018 | 40 | F | *C. parapsilosis* | GQ280289 | - | - | - |
|  | 21h | ZA019 | 40 | M | *C. metapsilosis* | GQ280290 | - | - | - |
| 36 | 36 | ZA020 | 28 | F | *Issatchenkia orientalis* | FJ697171 | - | - | - |
|  | 36b | ZA021 | 54 | F | *C. tropicalis* | JN606253 | - | - | - |
| 42 | 42 | ZA022 | 28 | F | *C. albicans* | JN606254 | 1605 | 4 | 8 |
|  | 42a | ZA023 | 28 | M | *C. albicans* | FJ662394 | 1609 | 4 | 8 |
|  |  | ZA023a |  |  | *C. albicans* | JN606255 | 659 | 4 | 8 |
| 143 | 143 | ZA024 | 50 | F | *C. albicans* | JN606256 | 1593 | 18 | 10 |
|  | 143a | ZA025 | 79 | F | *C. albicans* | FJ662403 | 1779 | 15 | 97 |
|  | 143b | ZA026 | 73 | F | *C. albicans* | FJ662401 | 1965 | 3 | 4 |
| 149 | 149 | ZA028 | 44 | F | *C. albicans* | - | 95 | 4 | 2 |
|  | 149a | ZA029 | 48 | M | *C. tropicalis* | FJ662409 | - | - | - |
| 152 | 152e | ZA030 | 78 | M | *C. tropicalis* | JN606259 | - | - | - |
| 157 | 157b | ZA031 | 56 | F | *C. parapsilosis* | FJ662413 | - | - | - |
|  | 157c | ZA032 | 56 | F | *C. albicans* | FJ662389 | 1061 | 4 | 2 |
|  |  | ZA032a | 54 | F | *C. albicans* | JN606260 | 144 | 4 | 2 |
| 159 | 159a | ZA033 | 64 | M | *C. parapsilosis* | FJ662414 | - | - | - |
| 163 | 163b | ZA034 | 6 | F | *C. albicans* | JN606261 | 1593 |  | 12 |
| 165 | 165 | ZA035 | 42 | M | *Lodderomyces elongisporus* | GQ280291 | - | - | - |
|  | 165a | ZA036 | 11 | F | *C. parapsilosis* | FJ662415 | - | - | - |
|  | 165b | ZA057 | 37 | F | *C. albicans* | GQ280292 | 1956 | 15 | 21 |
|  |  | ZA037 |  |  | *C. ethanolica* | FJ662418 | - | - | - |
|  | 165c | ZA038 | 70 | F | *C. tropicalis* | FJ662410 | - | - | - |
| 266 | 266b | ZA039 | 44 | M | *C. parapsilosis* | FJ662416 | - | - | - |
| 278 | 278b | ZA040 | 60 | F | *C. albicans* | FJ662402 | 1966 | 5 | 25 |
|  | 278c | ZA041 | 31 | M | *C. albicans* | FJ697169 | 1967 | 5 | 25 |
| 279 | 279a | ZA043 | 4 | M | *Meyerozyma guilliermondii* | FJ662408 | - | - | - |
|  | 279e | ZA044 | 54 | F | *C. parapsilosis* | FJ662417 | - | - | - |
| 281 | 281 | ZA045 | 32 | F | *Meyerozyma guilliermondii* | JN606264 | - | - | - |
| 287 | 287 | ZA046 | 29 | M | *C. albicans* | FJ662406 | 766 | 1 | 9 |
|  |  | ZA046a |  |  | *C. albicans* | JN606265 | 367 | 1 | 9 |
|  | 287a | ZA047 | 4 | F | *C. albicans* | FJ662396 | 656 | 4 | 2 |
|  | 287c | ZA048 | 27 | F | *C. albicans* | FJ662395 | 656 | 4 | 2 |
|  | 287g | ZA049 | 58 | F | *C. albicans* | FJ662390 | 656 | 4 | 2 |
|  |  | ZA049a |  |  | *C. albicans* | JN606266 | 142 | 4 | 2 |
| 289 | 289f | ZA053 | 45 | F | *C. albicans* | GQ280295 | 1593 | 18 | 10 |
|  | 289g | ZA054 | 67 | M | *C. albicans* | FJ662392 | 367 | 1 | 9 |

-, unknown

^a^ M, male; F, female

^b^ GenBank accession no. for the ITS1–5.8S–ITS2 region DNA sequences

^c^ DST, diploid sequence type
