# Supplementary material for: Particular Candida albicans Strains in the Digestive Tract of Dyspeptic Patients, Identified by Multilocus Sequence Typing
Source: PLoS One. 2012 Apr 20;7(4):e35311. doi: 10.1371/journal.pone.0035311 (PMC3335024; doi:10.1371/journal.pone.0035311)
Supplement: Table S2 — Details of the Candida positivity in the dyspeptic group and their family members. (DOC) [file pone.0035311.s002.doc]

**Table S2.** Details of the *Candida* positivity in the dyspeptic group and their family members

| Subject | age | sex^a^ | HP^b^ | diagnosis | Isolation source | isolate | species | ITS^c^ | DST^d^ | CLADE | eBURST  group |
| --- | --- | --- | --- | --- | --- | --- | --- | --- | --- | --- | --- |
| 422a | 57 | M | Y | superficial gastritis,  reflux esophagitis | oral | ZB001 | *C. albicans* | GQ280296 | 1957 | S | S |
| 429 | 35 | M | - | gastritis | oral | ZB002 | *C. albicans* | JN606271 | 1968 | 14 | S |
| 516a | 43 | M | Y | gastric ulcer,  atrophic gastritis,  duodenitis | oral | ZB005 | *C. albicans* | JN606272 | 142 | 4 | 2 |
| 703a | 58 | M | - | gastric cancer | oral | ZB007 | *C. albicans* | GQ280298 | 1593 | 18 | 10 |
| 703b | 56 | F | - | wife 703 | oral | ZB008 | *C. albicans* | GQ280299 | 1957 | S | S |
| 707 | 78 | F | Y | atrophic gastritis,  duodenal ulcer | gastric | ZB009 | *C. albicans* | GQ280300 | 1779 | 15 | 97 |
|  |  |  |  |  | oral | ZB010 | *C. parapsilosis* | GQ280301 | - | - | - |
| 709 | 39 | M | Y | gastric ulcer | gastric | ZB011 | *C. albicans* | GQ280302 | 1779 | 15 | 97 |
|  |  |  |  |  | oral | ZB012 | *C. parapsilosis* | GQ280303 | - | - | - |
| 709b | 37 | F | - | Wife 709 | oral | ZB013 | *C. albicans* | JN606277 | 1969 | 14 | 18 |
| 726 | 35 | M | Y | superficial gastritis,  duodenitis | oral | ZB015 | *C. albicans* | JN606278 | 1593 | 18 | 10 |
| 727 | 56 | F | N | atrophic gastritis | gastric | ZB016 | *C. albicans* | GQ280304 | 1593 | 18 | 10 |
|  |  |  |  |  | oral | ZB017 | *C. albicans* | GQ280305 | 1970 | 18 | 10 |
| 730 | 51 | F | Y | superficial gastritis | oral | ZB018 | *C. albicans* | JN606280 | 1593 | 18 | 10 |
| 803w | 49 | M | N | superficial gastritis | gastric | ZB020 | *C. albicans* | JN606281 | 1594 | 18 | 10 |
| 804w | 73 | M | Y | gastric ulcer,  duodenal ulcer | gastric | ZB021 | *C. albicans* | JN606282 | 1593 | 18 | 10 |
| 8011 | 38 | M | Y | gastric ulcer,  duodenal ulcer | oral | ZB023 | *C. albicans* | GQ280306 | 1593 | 18 | 10 |
| 8027 | 23 | F | Y | superficial gastritis | gastric | ZB024 | *C. albicans* | GQ280307 | 1971 | 18 | 10 |
|  |  |  |  | bile reflux |  | ZB024a | *C. albicans* | JN606284 | 1593 | 18 | 10 |
|  |  |  |  |  | oral | ZB025 | *C. albicans* | GQ280308 | 1958 | 18 | 10 |
|  |  |  |  |  |  | ZB025a | *C. albicans* | JN606285 | 1593 | 18 | 10 |
| 8062 | 30 | M | Y | superficial gastritis | gastric | ZB026 | *C. albicans* | GQ280309 | 1593 | 18 | 10 |
|  |  |  |  |  | oral | ZB027 | *C. albicans* | GQ280310 | 1593 | 18 | 10 |
| 8063 | 55 | M | Y | superficial gastritis,  bile reflux, | gastric | ZB028 | *C. albicans* | GQ280311 | 1593 | 18 | 10 |
|  |  |  |  | duodenal ulcer | oral | ZB029 | *C. albicans* | GQ280312 | 1593 | 18 | 10 |
| 8063a | 53 | F | - | Wife 8063 | oral | ZB030 | *C. albicans* | GQ280313 | 1593 | 18 | 10 |
| 8064 | 78 | M | Y | atrophic gastritis,  atypia dysplasia | oral | ZB031 | *C. albicans* | JN606288 | 1593 | 18 | 10 |
| 8066 | 42 | F | N | superficial gastritis | gastric | ZB032 | *C. albicans* | GQ280314 | 1594 | 18 | 10 |
|  |  |  |  |  |  | ZB032a |  | JN606289 | 1593 | 18 | 10 |
|  |  |  |  |  | oral | ZB033 | *C. albicans* | GQ280315 | 605 | 4 | 2 |
| 8081 | 38 | M | Y | gastric ulcer,  duodenal ulcer | gastric | ZB034 | *C. albicans* | JN606290 | 1971 | 18 | 10 |
|  |  |  |  |  | oral | ZB035 | *C. albicans* | GQ280316 | 1971 | 18 | 10 |
| 8082 | 30 | F | N | gastric ulcer | gastric | ZB036 | *C. albicans* | JN606291 | 1971 | 18 | 10 |
|  |  |  |  | atrophic gastritis | oral | ZB037 | *C. albicans* |  | 1971 | 18 | 10 |
| 8084 | 44 | F | Y | superficial gastritis | oral | ZB038 | *C. albicans* | JN606292 | 1959 | 14 | S |
| 8113 | 33 | F | N | superficial gastritis | gastric | ZB041 | *C. albicans* | JN606293 | 1593 | 18 | 10 |
|  |  |  |  |  | oral | ZB042 | *C. albicans* | JN606294 | 1972 | 7 | S |
|  |  |  |  |  |  | ZB042a | *C. albicans* |  | 1593 | 18 | 10 |
| 8115 | 45 | M | Y | atrophic gastritis | gastric | ZB043 | *C. albicans* |  | 1593 | 18 | 10 |
|  |  |  |  |  | oral | ZB044 | *C. albicans* | GQ280317 | 1973 | 4 | 2 |
| 8151 | 45 | M | Y | gastric ulcer | gastric | ZB045 | *C. albicans* |  | 1593 | 18 | 10 |
|  |  |  |  | duodenal ulcer | oral | ZB046 | *C. albicans* | GQ280318 | 1865 | 15 | 21 |
| 8152 | 44 | M | Y | superficial gastritis, | gastric | ZB047 | *C. albicans* | GQ280319 | 1593 | 18 | 10 |
|  |  |  |  | bile reflux | oral | ZB048 | *C. albicans* | GQ280320 | 1593 | 18 | 10 |
| 8184 | 65 | M | Y | atrophic gastritis, | gastric | ZB052 | *C. albicans* | GQ280321 | 1593 | 18 | 10 |
|  |  |  |  | intestinal metaplasia | oral | ZB053 | *C. albicans* | GQ280322 | 365 | 8 | 11 |
| 8185 | 71 | M | Y | superficial gastritis | gastric | ZB054 | *C. albicans* | GQ280323 | 1593 | 18 | 10 |
|  |  |  |  |  | oral | ZB055 | *C. albicans* |  | 1593 | 18 | 10 |
| 8186 | 40 | M | N | superficial gastritis | gastric | ZB056 | *C. albicans* | GQ280324 | 1593 | 18 | 10 |
|  |  |  |  | duodenitis | oral | ZB057 | *C. albicans* |  | 1593 | 18 | 10 |
| 8202 | 35 | F | N | superficial gastritis | oral | ZB058 | *C. albicans* | JN606297 | 1593 | 18 | 10 |
| 8203 | 31 | F | Y | superficial gastritis | gastric | ZB059 | *C. albicans* | GQ280325 | 1593 | 18 | 10 |
|  |  |  |  | bile reflux | oral | ZB060 | *C. albicans* | GQ280326 | 1974 | 14 | S |
|  |  |  |  |  |  | ZB060a |  | JN606299 | 1593 | 18 | 10 |
| 8205 | 18 | M | Y | superficial gastritis, | gastric | ZB061 | *C. albicans* |  | 1593 | 18 | 10 |
|  |  |  |  | bile reflux | oral | ZB062 | *C. albicans* | GQ280327 | 1593 | 18 | 10 |
| 8206 | 51 | M | Y | duodenal ulcer | gastric | ZB063 | *C. albicans* | JN606300 | 1593 | 18 | 10 |
| 8208 | 36 | M | Y | superficial gastritis,  duodenitis | gastric | ZB064 | *C. albicans* | JN606301 | 1593 | 18 | 10 |
| 8221 | 52 | M | Y | gastric cancer | oral | ZB065 | *C. albicans* | JN606302 | 1933 | 14 | 18 |
|  |  |  |  |  |  | ZB065a |  |  | 677 | 14 | 18 |
| 8224 | 57 | M | Y | gastric cancer | gastric | ZB066 | *C. glabrata* | FJ697172 | - | - | - |
| 8227 | 25 | F | Y | superficial gastritis,  bile reflux | gastric | ZB068 | *C. albicans* | GQ280328 | 1593 | 18 | 10 |
|  |  |  |  | duodenal ulcer | oral | ZB069 | *C. albicans* | GQ280329 | 1593 | 18 | 10 |
| 8229 | 62 | F | N | superficial gastritis | oral | ZB070 | *C. albicans* | JN606303 | 1975 | 7 | 17 |
| 8252 | 43 | F | N | superficial gastritis | gastric | ZB071 | *C. albicans* | GQ280330 | 1593 | 18 | 10 |
|  |  |  |  |  | oral | ZB072 | *C. albicans* | GQ280331 | 1593 | 18 | 10 |
| 8253 | 30 | M | Y | gastric ulcer,  bile reflux | gastric | ZB073 | *C. albicans* | GQ280332 | 1593 | 18 | 10 |
|  |  |  |  |  | oral | ZB074 | *C. albicans* | GQ280333 | 1593 | 18 | 10 |
| 8254 | 47 | M | Y | gastric ulcer | gastric | ZB075 | *C. albicans* | JN606304 | 1593 | 18 | 10 |
| 8291 | 63 | M | Y | superficial gastritis | oral | ZB078 | *C. albicans* | JN606305 | 1960 | 5 | S |
| 9034c | 35 | F | - | 9034 daughter | oral | ZB079 | *C. albicans* | JN606306 | 1871 | 8 | 11 |
| 9081 | 34 | F | Y | gastric ulcer,  duodenal ulcer | oral | ZB080 | *C. albicans* | JN606307 | 918 | 9 | 7 |
| 9084b | 70 | F | Y | gastric ulcer | oral | ZB081 | *C. albicans* | JN606308 | 747 | 4 | 40 |
| 9101b | 27 | F | N | superficial gastritis | gastric | ZB082 | *C. albicans* | GQ280334 | 601 | 12 | 6 |
|  |  |  |  |  | oral | ZB083 | *C. albicans* | GQ280334 | 601 | 12 | 6 |
| 9127 | 75 | M | N | atrophic gastritis | oral | ZB085 | *C. albicans* | JN606309 | 1976 | 4 | 2 |
| 9281 | 36 | F | - | superficial gastritis,  bile reflux | oral | ZB086 | *C. albicans* | JN606310 | 1977 | 4 | 8 |
| 9104 | 74 | M | Y | gastric cancer | oral | ZB088 | *C. albicans* | JN606311 | 1978 | S | 2 |

-, unknown

^a^ M, male ; F, female

^b^ Y, *H. pylori-*positive; N, *H. pylori-*negative

^c^ GenBank accession no. for the ITS1–5.8S–ITS2 region DNA sequences

^d^ DST, diploid sequence type
