# Supplementary material for: Particular Candida albicans Strains in the Digestive Tract of Dyspeptic Patients, Identified by Multilocus Sequence Typing
Source: PLoS One. 2012 Apr 20;7(4):e35311. doi: 10.1371/journal.pone.0035311 (PMC3335024; doi:10.1371/journal.pone.0035311)
Supplement: Table S3 — Details of the seven C. albicans gene loci for DST1593–1978. (DOC) [file pone.0035311.s003.doc]

**Table S3.** Details of the seven *C. albicans* gene loci for DST1593–1978

| strain | Original  no. | AAT1a | ACC1 | ADP1 | MPIb | SYA1 | VPS13 | ZWF1b | id | DST |
| --- | --- | --- | --- | --- | --- | --- | --- | --- | --- | --- |
| ZA014 | 19c | 5 | 32 | 10 | 6 | 7 | 74 | 5 |  | 1593 |
| ZB020 | 803w | 5 | 32 | 21 | 6 | 7 | 74 | 5 |  | 1594 |
| ZA003 | 2h | 13 | 7 | 15 | 6 | 7 | 32 | 12 | 2065 | 1961 |
| ZA004 | 2n | 8 | 14 | 8 | 4 | 7 | 100 | 22 | 2066 | 1962 |
| ZA008 | 13a | 3 | 3 | 5 | 3 | 57 | 10 | 6 | 2067 | 1963 |
| ZA010 | 13c | 33 | 32 | 5 | 2 | 156 | 202 | 8 | 2068 | 1964 |
| ZA026 | 143b | 13 | 10 | 15 | 6 | 7 | 37 | 212 | 2069 | 1965 |
| ZA032 | 157c | 8 | 7 | 8 | 4 | 2 | 10 | 8 | 2070 | 1061 |
| ZA040 | 278b | 4 | 3 | 48 | 18 | 31 | 111 | 15 | 2071 | 1966 |
| ZA041 | 278c | 4 | 26 | 48 | 18 | 31 | 32 | 15 | 2072 | 1967 |
| ZA057 | 165bl | 13 | 80 | 6 | 108 | 176 | 105 | 12 | 2060 | 1956 |
| ZB001 | 422a | 4 | 10 | 14 | 28 | 30 | 235 | 160 | 2061 | 1957 |
| ZB002 | 429 | 13 | 3 | 10 | 4 | 53 | 3 | 162 | 2073 | 1968 |
| ZB013 | 709b | 6 | 3 | 21 | 4 | 27 | 109 | 13 | 2074 | 1969 |
| ZB017 | 727 | 2 | 32 | 10 | 6 | 7 | 74 | 5 | 2075 | 1970 |
| ZB024 | 8027w | 5 | 32 | 21 | 6 | 82 | 74 | 5 | 2076 | 1971 |
| ZB025 | 8027 | 5 | 32 | 21 | 6 | 82 | 236 | 5 | 2062 | 1958 |
| ZB038 | 8084 | 6 | 3 | 21 | 4 | 38 | 237 | 13 | 2063 | 1959 |
| ZB042 | 8113 | 6 | 3 | 21 | 65 | 26 | 32 | 12 | 2077 | 1972 |
| ZB044 | 8115 | 14 | 14 | 6 | 4 | 7 | 10 | 8 | 2078 | 1973 |
| ZB046 | 8151 | 13 | 80 | 6 | 108 | 7 | 105 | 12 | 2079 | 1865 |
| ZB060 | 8203 | 2 | 3 | 21 | 50 | 27 | 109 | 5 | 2080 | 1974 |
| ZB065 | 8221 | 3 | 3 | 21 | 50 | 27 | 109 | 13 | 2081 | 1933 |
| ZB070 | 8229 | 6 | 3 | 37 | 2 | 26 | 32 | 12 | 2082 | 1975 |
| ZB078 | 8291 | 138 | 22 | 6 | 21 | 112 | 109 | 22 | 2064 | 1960 |
| ZB079 | 9034c | 55 | 14 | 4 | 3 | 6 | 45 | 12 | 2083 | 1871 |
| ZB085 | 9127 | 8 | 3 | 30 | 4 | 2 | 10 | 8 | 2084 | 1976 |
| ZB086 | 9281 | 11 | 3 | 6 | 4 | 34 | 60 | 119 | 2085 | 1977 |
| ZB088 | 9104 | 8 | 14 | 3 | 4 | 7 | 10 | 8 | 2086 | 1978 |

DST, diploid sequence type
